# Advances in High-Speed Structured Illumination Microscopy

**DOI:** 10.3389/fphy.2021.672555

**Published:** 2021-05-28

**Authors:** Tianyu Zhao, Zhaojun Wang, Tongsheng Chen, Ming Lei, Baoli Yao, Piero R. Bianco

**Affiliations:** 1 MOE Key Laboratory for Non-equilibrium Synthesis and Modulation of Condensed Matter, School of Physics, Xi’an Jiaotong University, Xi’an, China,; 2 State Key Laboratory of Transient Optics and Photonics, Xi’an Institute of Optics and Precision Mechanics, Chinese Academy of Sciences, Xi’an, China,; 3 College of Materials Science and Opto-Electronic Technology, University of Chinese Academy of Sciences, Beijing, China,; 4 MOE Key Laboratory of Laser Life Science & Guangdong Provincial Key Laboratory of Laser Life Science, College of Biophotonics, South China Normal University, Guangzhou, China,; 5 Department of Pharmaceutical Sciences, College of Pharmacy, University of Nebraska Medical Center, Omaha, NE, United States

**Keywords:** fluorescence microscopy, super-resolution, SIM, hardware acceleration of deep learning, image reconstructed algorithm

## Abstract

Super-resolution microscopy surpasses the diffraction limit to enable the observation of the fine details in sub-cellular structures and their dynamics in diverse biological processes within living cells. Structured illumination microscopy (SIM) uses a relatively low illumination light power compared with other super-resolution microscopies and has great potential to meet the demands of live-cell imaging. However, the imaging acquisition and reconstruction speeds limit its further applications. In this article, recent developments all targeted at improving the overall speed of SIM are reviewed. These comprise both hardware and software improvements, which include a reduction in the number of raw images, GPU acceleration, deep learning and the spatial domain reconstruction. We also discuss the application of these developments in live-cell imaging.

## INTRODUCTION

Fluorescence microscopy is a powerful tool for visualizing biological processes of molecules and intracellular structures in living cells. However, due to the diffraction limit, fine structures within organelles whose dimension is less than ~200 nm laterally and ~500 nm axially are difficult to observe [1]. Over the past several years, exponential growth in using super-resolution (SR) fluorescence microscopy has occurred in the field of biomedical imaging [2]. SR techniques have shattered the diffraction limit, thus researchers are able to investigate fine details of biological structures at the nanometer level. These imaging systems include stimulated emission depletion (STED) microscopy [3–6], photo-activated localization microscopy [7–9], stochastic optical reconstruction microscopy [10, 11], and structured-illumination microscopy (SIM) [12–14] Among these approaches, SIM has attracted considerable interest because of its low light dose and high imaging speed [15]. These characteristics provide a powerful tool in various biological applications including cortical microtubules in Arabidopsis, spine morphology [16], neurotrauma [17], and macrophages in the ischemic tissue [18].

In SIM, the periodic illumination down-modulates the high spatial frequency of the sample information in the Fourier domain. Several raw images with different pattern phases and orientations are captured to undo the frequency modulation. The final high-resolution image is computed after separating and recombining the spectral information [19, 20]. Unfortunately, the capturing of multiple images and post-processing of these images slows down the speed of SIM, resulting in difficulties of SR imaging in real-time *in vivo* [21]. To circumvent these issues, several groups have implemented modifications designed to increase the overall imaging speed of SIM. In this review, we first introduce the basic principle of SIM. Then we focus on the optical configuration of a fast SIM system and compare the different fast SIM implementations that include optimization of the optical configuration as well as improvements in image reconstruction methods.

## THE THEORETICAL BASIS OF SIM

**FIGURE 1 |:**
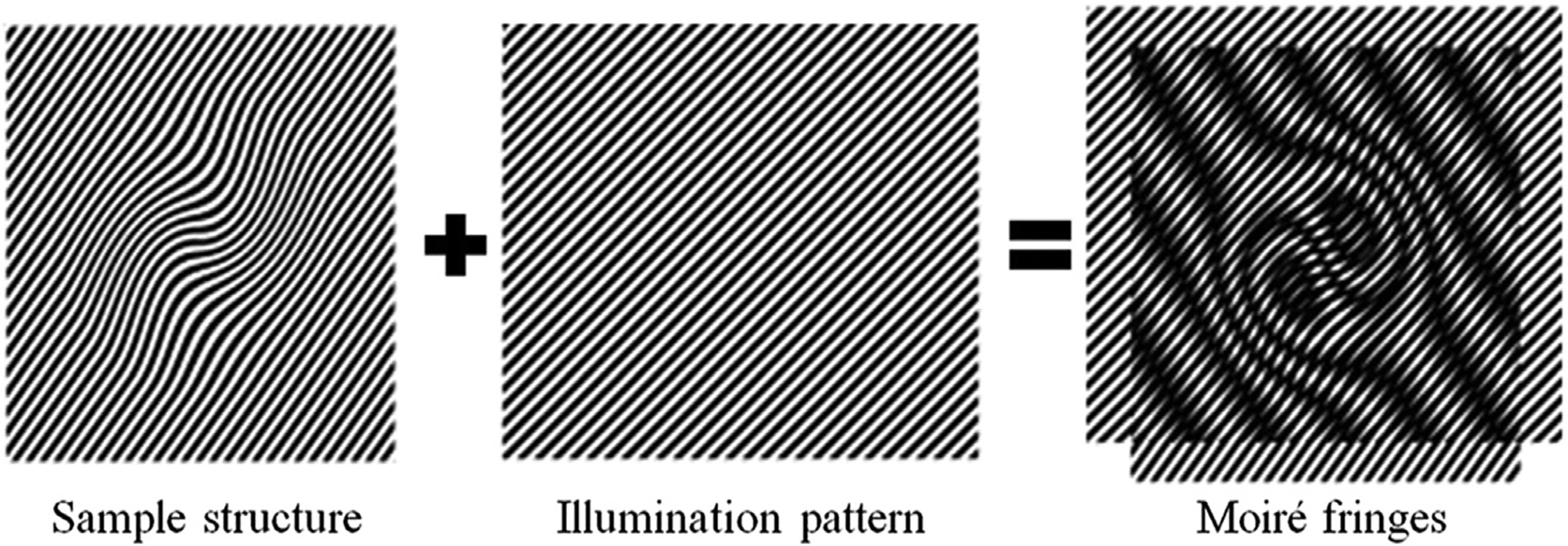
Generation of Moiré fringes. If the sample structure is multiplied by structural light, Moiré fringes will appear.

The concept behind SIM can be easily understood in terms of the well-known moiré effect. If two fine patterns are superposed multiplicatively, a beat pattern will appear in their product (Figure 1). In the frequency domain, there is a cut-off frequency in optical microscopy. The high-frequency information of the sample light is lost after passing through an objective lens, which leads to the diffraction limit of conventional optical microscopy. If the microscope can collect high spatial frequency information, closely spaced molecules can be resolved from each other. In traditional optical microscopy systems, the spatial resolution of the microscope objective depends on the maximum spatial frequency $f_{0}$ it can collect. In SIM, the sample is illuminated by the modulated light with a spatial frequency $f_{1}$. Thus, the detector receives the moiré fringe including the spatial frequency $\left|f-f_{1}\right|<f_{0}$. Because of the moiré effect, high-frequency information moves into the observable region, which is normally unresolvable using conventional microscopy. By resolving the high-frequency information in the moiré fringe, the high-frequency information can be computed, and the resolution enhanced.

Considering a specimen with the fluorescent molecular distribution density S(r) and illumination light I(r), the image collected by the camera D(r) is the convolution of the fluorescent and the point spread function H(r) [22].

(1) $$D\left(r\right)=\left[I\left(r\right)\cdot S\left(r\right)\right]⊗H\left(r\right),$$

Where r is the spatial coordinates. In SIM, the illumination is a cosine fringe intensity pattern I(r) with the form

(2) $$I(r)=[1+m\cdot \text{cos}(2\pi p\cdot r+\varphi )]\cdot I_{0},$$

Where m, p, φ, and I0 are the modulation depth, the spatial frequency, the initial phase of the cosine fringe pattern and the mean intensity, respectively. After combining Eqs 1, 2, the spectrum of the detected image in the frequency domain can be obtained by taking the Fourier transform

(3) $$\tilde{D}(k)=I_{0}\left[\tilde{S}(k)+\frac{m}{2}\tilde{S}(k+p)e^{-i\varphi }+\frac{m}{2}\tilde{S}(k-p)e^{i\varphi }\right]\cdot \tilde{H}(k),$$

Where $\tilde{S}(k)$ and $\tilde{H}(k)$ are the Fourier transform of the fluorescent distribution and the optical transfer function (OTF), and k is the frequency coordinates. Eq. 3 shows that the high-frequency features of the sample $\tilde{S}(k+p)$ and $\tilde{S}(k-p)$ are shifted into support areas of the detection OTF by structured light. As the result of diffraction effect, the cut-off frequency $k_{0}$ limits the spatial frequency of the system k as $k\in \left[-k_{0},k_{0}\right]$ and is enlarged by the structured illumination into $k\in \left[-k_{0}-p,k_{0}+p\right]$. Also, since the illumination pattern is generated through the objective lens, the illumination pattern is diffraction-limited as $p\leq k_{0}$. Thus the maximum spatial frequency of SIM is $k\in \left[-2k_{0},2k_{0}\right]$, which means that the maximum resolution enhancement in SIM is two-fold over that of the traditional microscopy [23].

To separate the high-frequency component in the superposing frequency spectrum, three frequency spectrum components $\tilde{S}(k)$, $\tilde{S}(k+p)$ and $\tilde{S}(k-p)$ should be solved. The most usual method is to capture three raw images with different phase shifts between each other and solve the equations as

(4) $$\left[\begin{array}{l} {\tilde{D}}_{1}(k)\\ {\tilde{D}}_{2}(k)\\ {\tilde{D}}_{3}(k) \end{array}\right]=I_{0}\tilde{H}(k)\left[\begin{array}{ccc} 1 & \frac{m}{2}e^{-i\varphi_{1}} & \frac{m}{2}e^{i\varphi_{1}}\\ 1 & \frac{m}{2}e^{-i\varphi_{2}} & \frac{m}{2}e^{i\varphi_{2}}\\ 1 & \frac{m}{2}e^{-i\varphi_{3}} & \frac{m}{2}e^{i\varphi_{3}} \end{array}\right]\left[\begin{array}{c} \tilde{S}(k)\\ \tilde{S}(k+p)\\ \tilde{S}(k-p) \end{array}\right]$$

Finally, based on the amplitude and offset of the illumination pattern determined above, the three frequency spectrum components are moved to the correct position and the merged [24]. In order to enhance the isotropic resolution, other images with multiple illumination directions are also required. For example, for 2D SIM, three directions × three phase shifts are needed, which means there are a total of nine original images.

## OPTICAL CONFIGURATION FOR FAST SIM

**FIGURE 2 |:**
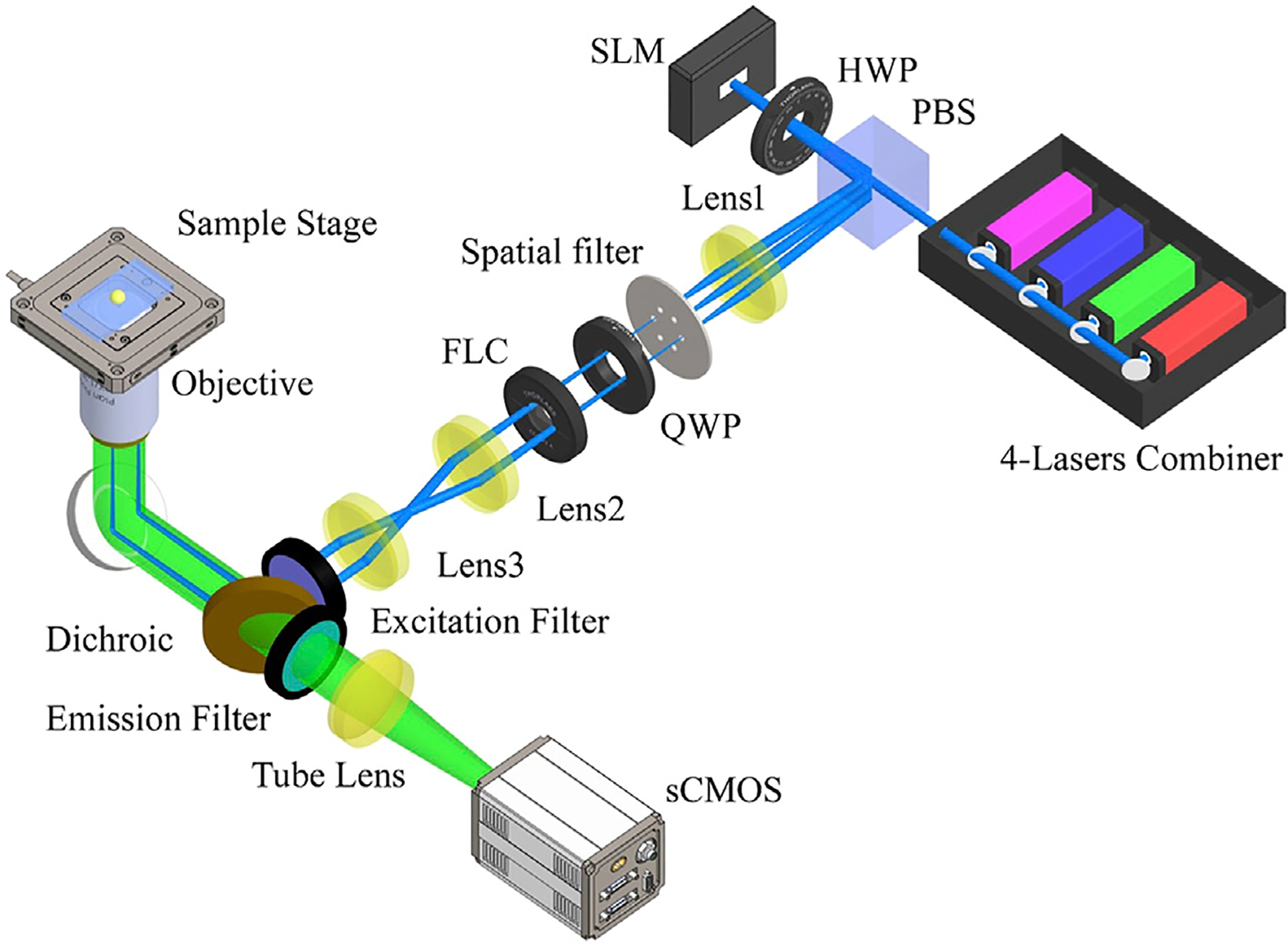
Schematic of a high-speed SIM system. PBS, polarization beam splitter; HWP, half-wave plate; FC-SLM, ferroelectric liquid crystal spatial light modulator; QWP, quarter-wave plate; FLC, ferro-electric liquid crystal phase retarders. The layout is similar to the original SIM presented in [15] but the hardware components have changed over the years.

A basic SIM setup consists of three main elements: the generation of structured light, polarization control, and detection. In common SIM, the interference of multiple laser beams generates a sinusoidal illumination pattern to illuminate samples, which is achieved using a typical, optical layout shown in Figure 2. Based on this setup, we introduce several improvements for fast SIM in the following section. In the setup, the expanded and collimated laser beam is modulated by a ferroelectric liquid crystal spatial light modulator (FC-SLM). The laser beam is divided by a diffraction grating generated by the FC-SLM. The ±1 order diffraction beams for 2D-SIM or, the ±1 and 0 order beams for 3D-SIM are selected by a spatial filter. After adjusting the polarization, the laser passes through a 4f system and is focused by the objective. The ±1-order diffraction beams interfere at the focal plane to produce sinusoidal patterns on the specimen. A dichroic mirror reflects the emitted fluorescence signal which is then collected by an sCMOS camera.

### Generation of Structured Light

In traditional SIM, cosinoidal illumination is generated by the interference of multiple laser beams. The fringe pattern generation and phase shift control are directly related to the SR image, which are the core technologies of SIM. In 1999, the first generation of SIM used a transmission grating with mechanical devices for moving and rotating [25]. The mechanical movement of the grating was slow and limited the acquisition speed to several seconds per SIM image.

**FIGURE 3 |:**
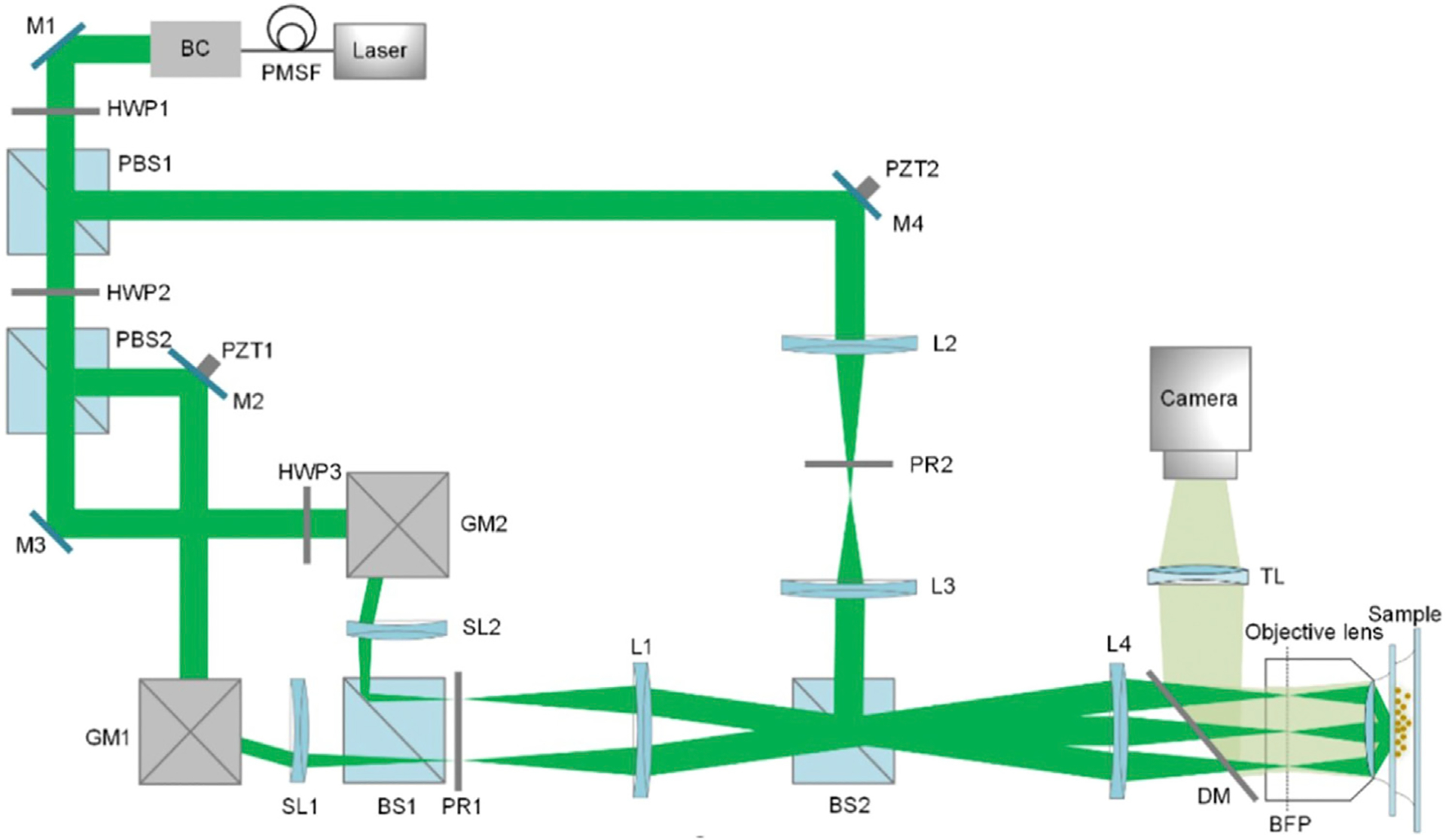
The diagram of 3D galvanometer-based SIM [31]. PMSF, polarization-maintaining single-mode fiber; BC, beam collimator; PBS, polarized beam splitter; GM, scanning galvanometer; PZT, piezoelectric stage; SL, scanning lens; PR, polarization rotator; BFP, back focal plane; DM, dichroic mirror; TL, tube lens. The figure is modified with permission from Ref. (31). Copyright ^©^ 2019 The Optical Society.

To improve this, the FC-SLM replaced the diffraction grating. The wavefront of the input light is modulated by changing the liquid crystal alignment of the FC-SLM with the desired illumination pattern being generated in less than 1 ms [26]. Consequently, the switching between grating transfers and orientations, which occurs at speeds more than 10 times faster than a motor-based system, greatly improves the temporal resolution of SIM. By using an FC-SLM, Kner et al. and Shao et al. achieved SR imaging of biological samples at ~100 nm resolution at frame rates of 11 and 0.2 frames per second (fps) in 2D- and 3D-SIM, respectively [27, 28]. However, replacing a physical grating with a pixelated SLM has some trade-off. Because of introduced additional unwanted diffraction orders by the SLM display, there are jagged edges in the illumination pattern and loss of laser power. The special filter can block the unwanted frequency spectrum and form a cosinoidal illumination grating, at the expense of light efficiency [29, 30]. A third way to generate the structured light is by scanning the sample with galvanometers (Figure 3). Using this approach, an entire U2OS cell with a field of view greater than 40 μm *40 μm was recorded in 4 s at 10 fps [31, 32]. However, this system is more complex than the system shown in Figure 2 and requires precise adjustment [33].

### Polarization Control

To obtain optimal contrast of the fringe pattern in the focal plane of the objective, the polarization of the two interference beams needs to be carefully adjusted [34, 35]. Theoretically, the ±1-order diffraction beams should both be s-polarized to reach the maximum contrast in the fringe pattern [36]. Moreover, SIM imaging requires illumination patterns along at least three different orientations to ensure even resolution improvement. The polarization states of the incident beam also need to be adjusted when switching the orientations of illumination patterns.

**FIGURE 4 |:**
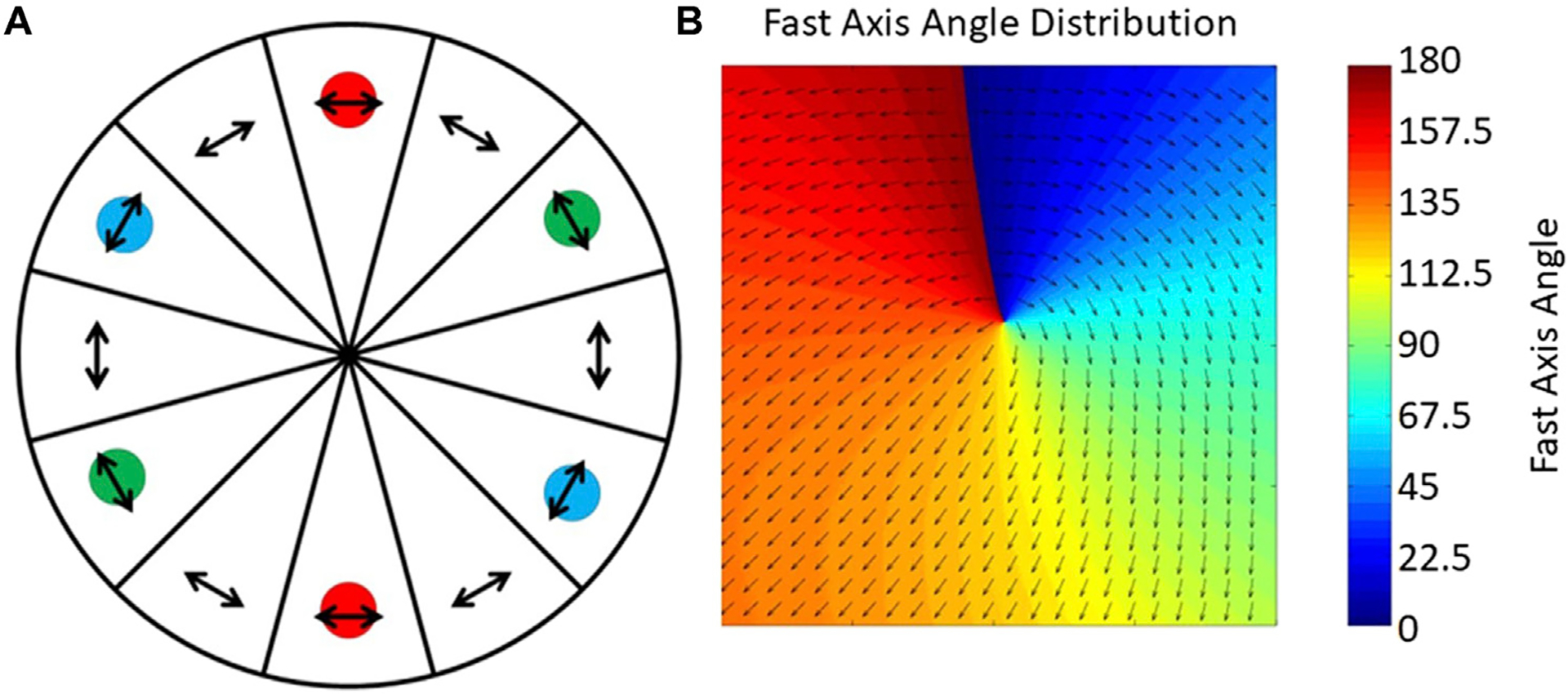
Unique approaches to improving polarization control. **(A)**, The pizza polarizer. The arrows indicate the direction of the transmission axis of each polarizer and the color circles indicate the position of ±1-order diffraction beams on the pizza polarizer [37]. The figure is modified with permission from Ref. (37). Copyright ^©^ 2015 IOP Publishing. **(B)**, The zero-order vortex half-wave retarder is a non-uniform half-wave plate whose fast axis distributes in the direction of the arrows shown [39].

Initially, a mechanical rotator with a half-wave plate was used to control polarization, but this method is both slow and unstable [15]. Nine years later, two ferro-electric liquid crystal phase retarders (FLC) were employed to rotate the polarization of the light and illumination pattern synchronously. However, FLCs have a switching time of over 20 ms, which limits the SIM imaging speed to around 50 ms per frame. To avoid the switching time of the FLCs, a passive polarization control scheme was presented where the FLCs were replaced with a quarter-wave plate and a pizza polarizer, which is a custom-made, twelve fan-shaped polarizer (Figure 4A) [37, 38]. Unfortunately, the pizza polarizer, which requires an experienced manufacturing setup, causes a 50% reduction in laser power. More recently, Zhao et.al improved this method by making use of the zero-order vortex half-wave retarder, which is a non-uniform half-wave plate whose fast axis distributes as the arrows shown in Figure. 4B [39, 40]. It is efficient but only suitable for a single wavelength, which limits its application in multi-color imaging [41].

### Detection

For fast SIM systems, sCMOS cameras have significant advantages over EMCCD or CCD cameras regarding field-of-view size and readout speed [42, 43]. When the limiting factor of the system is the camera readout speed, sCMOS detectors combined with a rapid illumination pattern generator like FC-SLM is the superior choice for fast and live-cell acquisition. However, the FC-SLM updates in a synchronous manner, whereas the camera used by fast structured illumination systems usually reads the imaging data out in an asynchronous way as a result of rolling shutter mode. This leads to synchronization problems thus limiting the acquisition speed of fast SIM.

**FIGURE 5 |:**
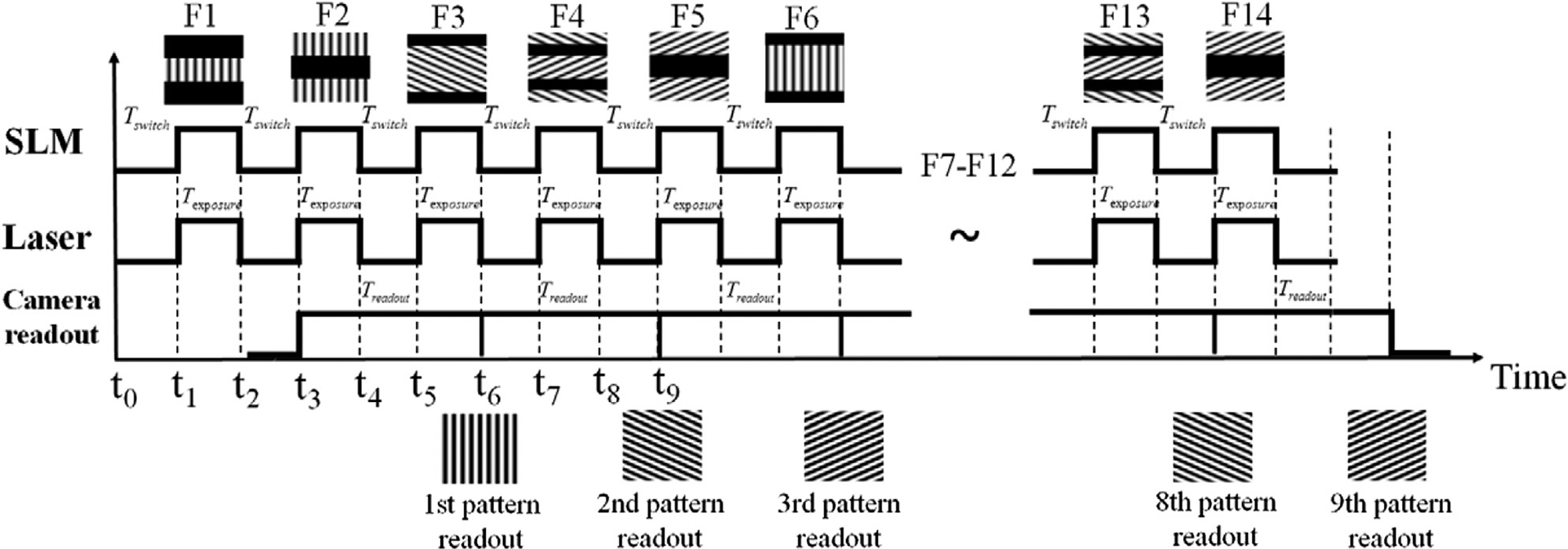
The modified synchronization configuration corresponding to segmented frames [44]. When the camera starts the rolling shutter from the middle of the frame at time t_0_, the first segmented F1 is initially uploaded into the SLM. After the SLM finishes uploading F1 at t_1_, the laser begins to illuminate for $T_{\text{exposure}}$ and the camera detects the emission from the illumination pattern F1. At t_2_ the laser is off for $T_{\text{switch}}$ and the SLM starts to upload the second frame F2. The camera readout starts at t_3_ and the first raw frame receives at t_6_. The figure is modified with permission from Ref. (44). Copyright ^©^ 2016 IOP Publishing.

To address this, Song *et al* presented a configuration which displayed multiple SLM frames per camera readout cycle by dividing the extremely fast SLM display into several segments along the direction of the rolling shutter of the sCMOS camera (Figure 5) [44]. Here, a series of specially designed patterns are used to coordinate the sCMOS. By presenting different SIM patterns during the start exposure and readout line, the SLM maintains the readout line of the camera inside the dark region when the camera runs in continuous rolling shutter mode. This approach reached an acquisition rate of 79 fps with the raw frame rate of 714 fps, the field of view at 16.5 μm × 16.5 μm and the laser illuminating time of 0.5 s.

### Adaptive Optics

As with all microscopes, aberrations also detrimentally affect imaging in SIM, resulting in a loss of resolution due to spherical aberration while imaging thick samples [45]. Image quality can be restored using the techniques of adaptive optics (AO), where wavefront sensing and adaptive elements, such as a deformable mirror (DM) or SLM, are used to correct the depth-dependent aberrations [46]. To overcome the aberration and imaging at depth, AO have been an attractive line of research in SIM. The combination of AO and SIM was applied to image through 35 μm of *C. elegans* samples with a resolution of 140 nm [47]. Although the complex adjustment by AO devices takes typically 2–3 min, a one-time wavefront distortion measurement and correction ensure high-quality, hours-long recordings [48]. More recently, Turcotte et al. reported an application of AO to correcting sample-induced optical aberrations *in vivo* with 9.3 fps at a depth of 50 μm, demonstrating the application of SIM in live tissue and *in vivo* imaging [49].

### Summary

To permit direct comparisons, we present a comparison flowchart of SIM systems, with more details of the key components of these methods and their performance summarized in Table 1. The reader should note that a combination of these methods is feasible, in which the frame rate of SIM images can reach up to 188 fps [50]. However, it will significantly increase the difficulty of building the system. The skillful work by Justin et al [51] and software like SIMcheck [52] provide advanced calibration tools and utilities to create an optimal SIM imaging environment.

## RECONSTRUCTION METHODS FOR FAST SIM

### Fewer Raw Images

To unmix spatial frequencies along a single direction, conventional 2D-SIM requires three images to be acquired with translated illumination patterns [53]. In order to provide isotropic resolution enhancement, SIM needs to perform this process three times and rotate illumination fringes three times, which yields a total of nine raw images per SR SIM image [54]. Acquiring these nine images to yield a single super-resolution image can be time-consuming. Consequently, reducing the number of raw frames required to produce a SIM image has been an active field of research. It has the potential to increase the acquisition speed and reduce phototoxic effects and bleaching.

In Eq. 3, the $\tilde{S}(k)$, $\tilde{S}(k+p)$ and $\tilde{S}(k-p)$ are not independent on one another. This means less than three raw images of a single direction can solve the spatial frequencies and less than nine raw images should be enough for SIM reconstruction. In 2003, Heintzmann realized the redundant data in the SIM algorithm and discussed the potential for substantially decreasing the required number of raw images in SIM. However, Eq. 4 cannot be solved directly under the condition of fewer raw data, and newer methods were required [55]. In 2015, an alternative imaging strategy was presented, which reduced the number of image acquisitions into four raw images and reconstructed the SR image by a modified, incoherent Fourier ptychographic procedure [56]. Because of the properties of the Fourier transform, there is same information in the ±1 order of the Fourier spectrum of raw images. 0 and π phase shifts illumination fringes can be simply used as the modulating pattern. Thus the method first uses two commentary patterns with the same direction with 0 and π phase shifts, providing a uniform modulation for the sample. A good initial guess is formed by the summation of these two images for the high-resolution sample image. The other two patterns provide different orientations to isotopically double the bandwidth in the Fourier domain. To recover the SR image from the four raw images, the Fourier ptychographic needs the initial guess and the target image as

(5) $$I_{\text{obj}}=I_{1}+I_{2},\;I_{tn}=I_{\text{obj}}\cdot P_{n}$$

where $I_{n}(n=1,2,3,4)$ is the raw images and $P_{n}(n=1,2,3,4)$ is the illumination sinusoidal patterns. Then the initial guess and the target image updates with $I_{n}$ as

(6) $$I_{\text{tn}}^{\text{updated}}=I_{\text{tn}}+\text{deconvwnr}\left(I_{n}-I_{\text{tn}}⊗H\right),$$

(7) $$I_{\text{obj}}^{\text{updated}}=I_{\text{obj}}+\frac{P_{n}}{{\left(\text{max}\left(P_{n}\right)\right)}^{2}}\left(I_{\text{tn}}^{\text{updated}}-I_{\text{obj}}\cdot P_{n}\right),$$

where H, deconvwnr and max stand for the point spread function, wiener deconvolution and taking the maximum value, respectively. Eqs 6, 7 are iteratively repeated for the four measurements. The iteration process is terminated until the difference between two successive recoveries is less than the given limit of error. Although this 4-frame SIM method decreases the data acquisition time greater than two-fold, analysis of each area of the spectra in SIM revealed that there is still an overdetermined equation system in this approach. To overcome this, the authors proposed an underdetermined SIM with three raw images and generated an estimate of the SR image by using a joint Richardson-Lucy deconvolution algorithm [53]. Therefore, it needs only one-third acquiring time compared to the traditional method.

However, both 4-frame SIM and 3-frame SIM utilize iterative algorithms to arrive at the reconstructed image, whereas the conventional 9-frame SIM provides a single-step solution. Over 200 loop numbers are needed to converge the SR image, which leads to a significant increase in the amount of time required for post-processing.

### GPU Acceleration and Real-Time Display

To reduce the effects of movement in the sample and or the microscope system, image capturing, processing, and the SR image display must occur in rapid succession when imaging live-cells in real-time [57]. The datasets are normally post-processed to speed up the capturing process due to the time-consuming image reconstruction in computational processing. This dilemma has limited the use for real-time evaluation of samples since SR images are not available during image acquisition [58].

To realize the real-time reconstruction, Markwirth et al. utilized a graphics processing unit (GPU) to perform high-speed image reconstruction [59]. GPUs take advantage of executing calculations in a parallel fashion dealing with SIM reconstruction, which is faster than the serial fashion on computer central processing units (CPUs). This follows because the CPU executes calculations sequentially, significantly slowing down the process of images. Using the GPU, a more than 10 fold increase in processing speed was achieved comparing with processing on a multi-core CPU [60].

**FIGURE 7 |:**
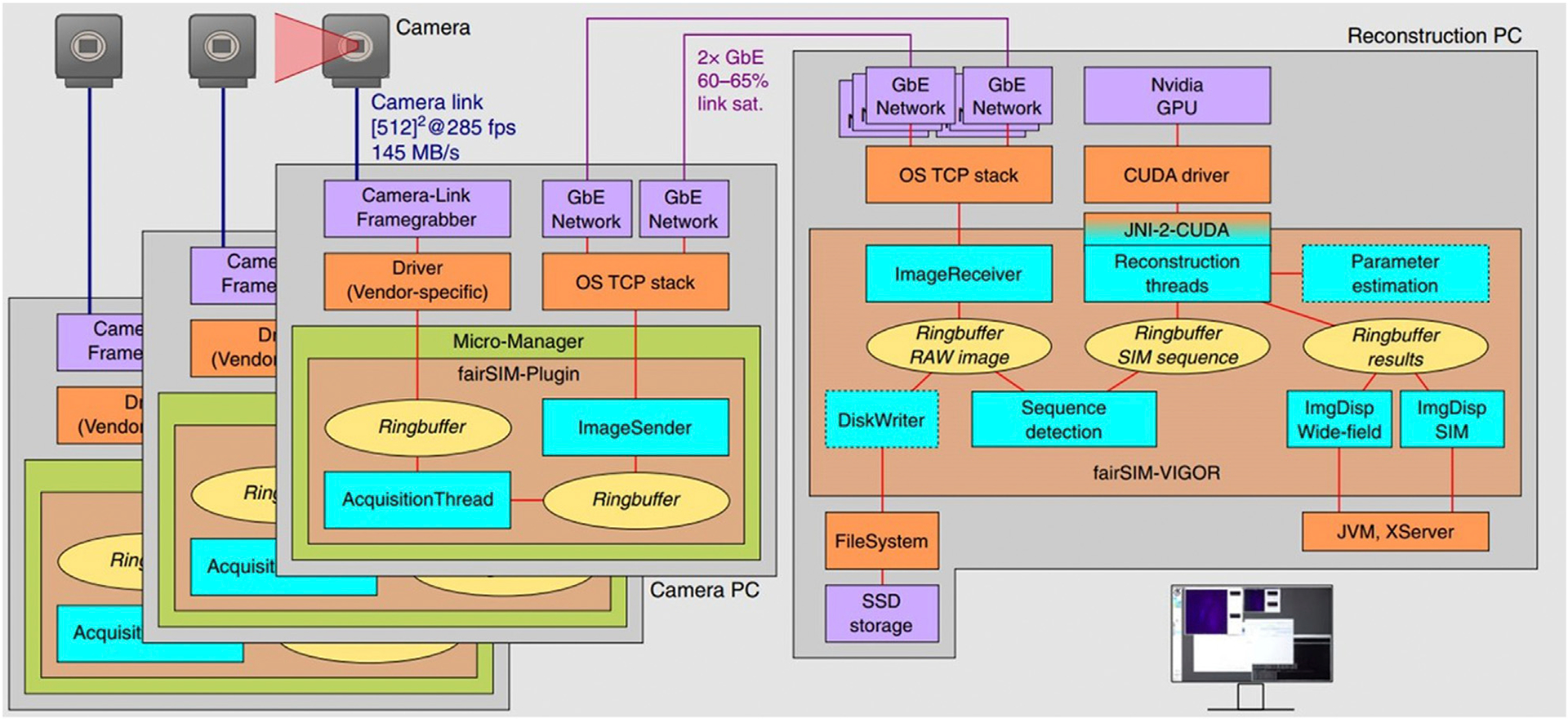
Structure of the real-time reconstruction software fairSIM-VIGOR [62]. These schematics show the data flow of the raw images through the on-the-fly reconstruction pipeline, which from sCMOS to Camera PC is on the left part of the figure (note that there are three of these systems working simultaneously) and from Camera computers to Reconstruction PC is on the right part of the figure. The left part of the image shows the data flow within each camera PC and the right part is flow through the reconstruction PC. The figure is modified with permission from Ref. (62). Copyright ^©^ 2019 Springer Nature.

To display the reconstruction results in real-time, it is necessary to increase the data transmission and processing speed [61]. Continuous reconstruction is achieved by a modularized, networked, and multi-threaded framework (Figure 7) [62]. Here, three cameras, each connected to a separate computer were used to capture images (one per laser wavelength). In order to permit the rapid transfer of camera data for nearly instantaneous processing on the GPU, these three systems were connected to a dedicated reconstruction computer by Gigabit Ethernet lines.

It takes ~200 ms to reconstruct a 2D SIM for a typical input image size of 512 × 512 pixels using a current multi-core desktop CPU. Including data transfer to the GPU and back, there is about 20 ms latency to the image processing pipeline in the GPU-assisted SIM reconstruction on a mid-range consumer-level graphics card (Nvidia GTX 1060). Experimental results show 10.4 fps SR imaging speed and less than 250 ms delay between measurement and reconstructed image display, with 512 × 512 pixels and 2 ms illumination time per raw frame. While this approach demonstrates the advantage of GPU-processing, the drawback is the complicated and expensive system which has multiple cameras each with a computer and a fourth PC performing reconstruction.

### Deep Learning

Deep learning is a kind of machine learning, which is a powerful tool for increasing the depth of the neural network. It emphasizes looking for increasingly meaningful representations during learning from successive layers [63]. Deep learning methods have been applied to SR microscopy in recent years and could be a solution for high-speed SIM. After training by hundreds of paired training data, the deep learning framework can produce the SR image from fewer raw images and boost the performance of the SIM under low-light conditions. This has the potential to allow for reducing phototoxicity in the imaging process and obtaining the SR imaging at higher speeds [64].

**FIGURE 8 |:**
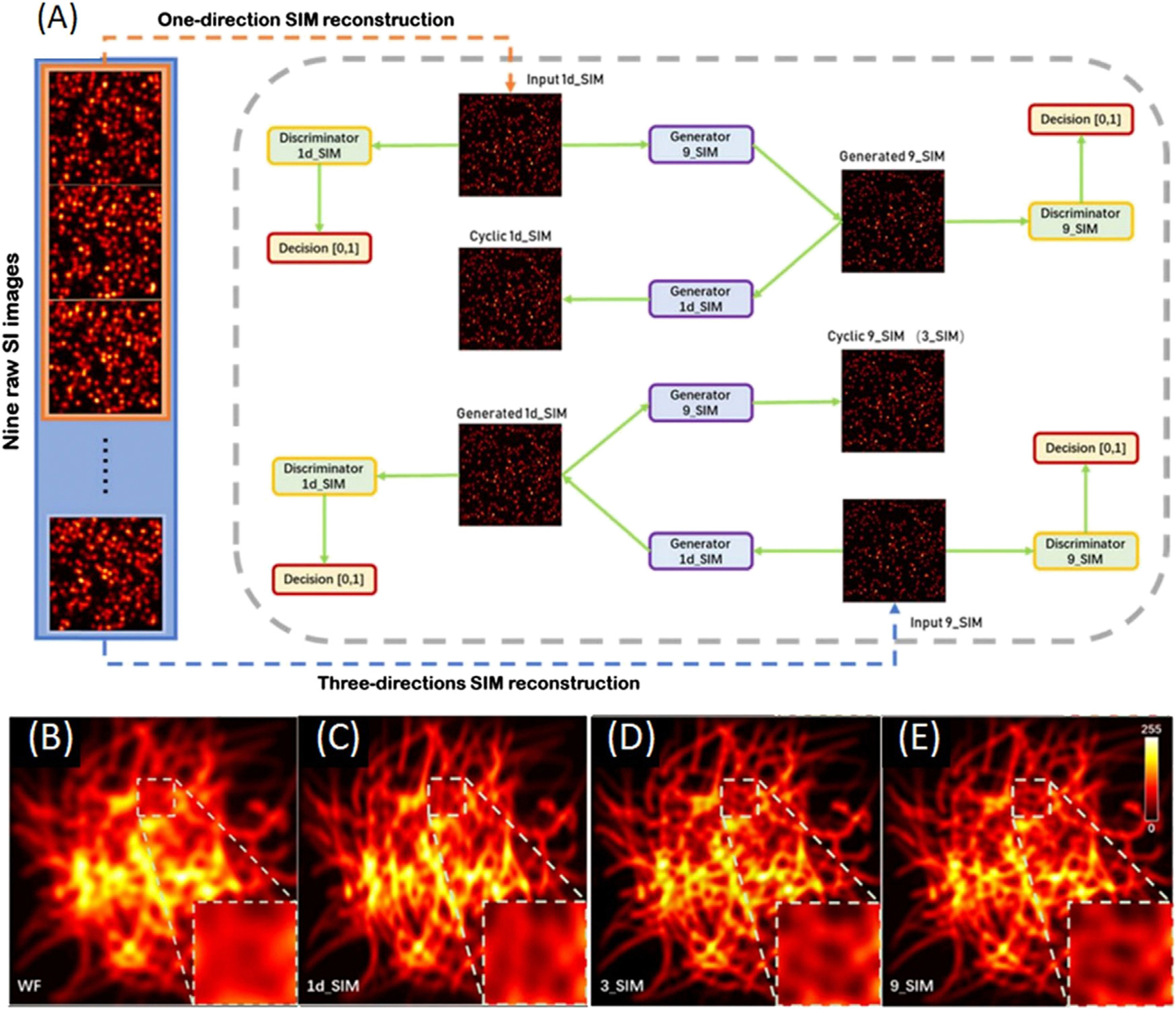
Schematics and results of the deep neural network trained for SIM imaging [65]. **(A)** Deep neural network training cycles. Two training datasets are 1d_SIM and 9_SIM images as the input of two training cycles, following with two generators and two discriminators. These generators and discriminators are trained by optimizing various parameters. This loops until the generated images are accepted by the discriminator. **(B-E)** Deep learning-enabled transformation of images from 1d_SIM to 9_SIM. The 3_SIM image generated by the deep neural network in **(D)** is matched to the 9_SIM image in **(E)**. Figures are modified with permission from Ref. (65). Copyright ^©^ 2020 Chinese Laser Press.

To illustrate how this works, one example of a deep neural network trained for SIM imaging is shown in Figure 8 [65]. Two datasets are needed for training the neural network, which are the SR image in one direction as train A (1d_SIM) and the SR image in three directions as train B (9_SIM). There are two generators and two discriminators featured the deep neural network. Generator 9_SIM transformed images in train A into generated 9_SIM and generated 9_SIM images are transmitted to cyclic 1d_SIM by generator 1d_SIM. Same process subjects to the input images of the 9_SIM dataset. The input of discriminator A and discriminator B are images of the 1d_SIM and 9_SIM datasets, respectively. The loss function is used to train the data, identifying if the output image is generated by the generator or the raw image. This process is repeated until the generated images are accepted by the discriminator. Experimental results in Figures 8D,E show that only three raw images in a given direction can generate an SR image in three directions and the resolution of the reconstruction rivals that of the traditional SIM methods.

### Reconstruction in the Spatial Domain

The inventor of SIM, Mats G. Gustafsson once said: “I like to think in frequency space, rather than in real space” [66]. Consequently, in traditional SIM systems, the basic workflow to generate the SR image is built on spatial spectrum processing and Fourier domain reconstruction (FDR). The transfer time between spatial and frequency domain slows down the reconstruction speed of SR images inevitably [67, 68]. Since the frequency and spatial domains can be transformed into each other, it should be possible to reconstruct the SR image in the spatial domain. This concept appeared in early works where the theoretical framework was laid down [69, 70]. Recently, the ability to reconstruct the SR image in the spatial domain was demonstrated experimentally by a significantly improved algorithm which is called spatial domain reconstruction or, SDR [71].

The mathematical background of SDR can be described from the perspective of point-spread-function (PSF) engineering, which in SIM generates a composite image with an effective PSF narrower than conventional wide-field microscopy [70]. Thus, the SR image $R_{\text{SDR}}(r)$ and its PSF P(r) can be described as

(8) $$R_{\text{SDR}}\left(r\right)=S\left(r\right)⊗P\left(r\right),$$

(9) $$P\left(r\right)=\left[1+\text{cos}\left(2\pi p\cdot r\right)\right]\cdot H\left(r\right).$$

In the SDR scheme, the patterned illuminated raw images are linear superposition with appropriately weighted coefficients c to attain the SR image. Then the reconstructed SR image is

(10) $$R_{\text{SDR}}(r)=\sum_{\text{ja}1}^{n}c_{j}(r)[I(r)\cdot S(r)]⊗H(r),$$

where j indicates the several steps shifting of the illumination pattern. It is solvable to the expressions of $c_{j}(r)$ after expanded into a series of orthogonal complete basis and substituting Eq. 10 into Eq. 8.

**FIGURE 9 |:**
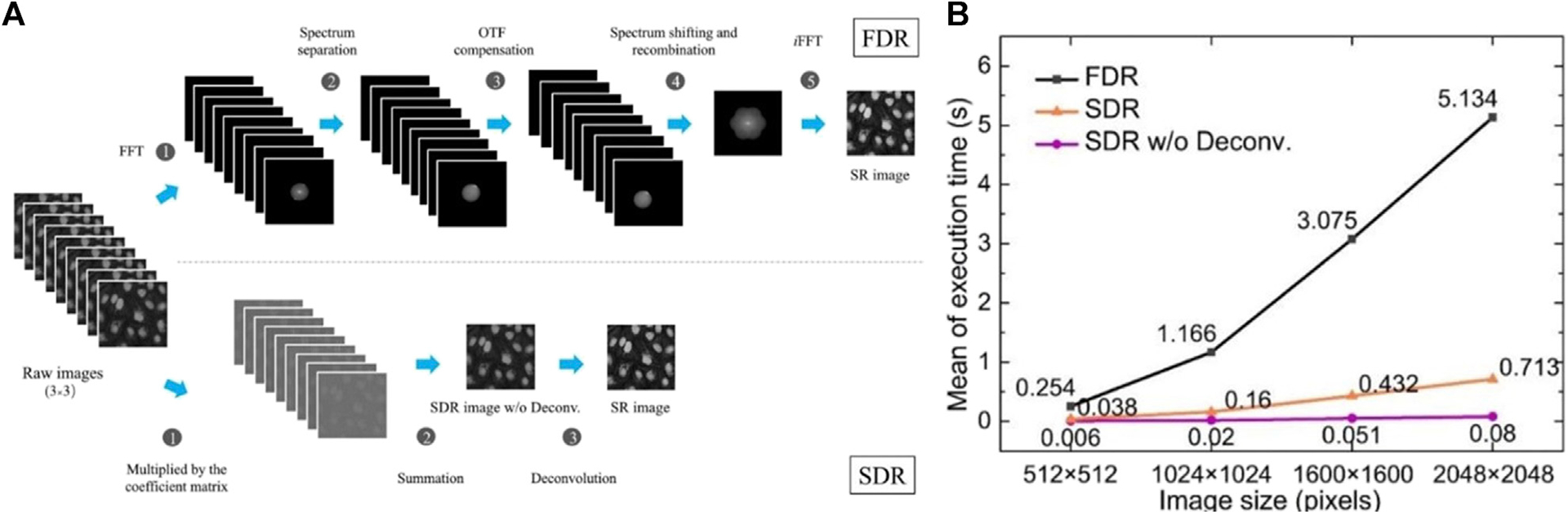
SDR generates SR images 64-fold faster than FDR [71]. **(A)** The comparison of the FDR and SDR image reconstruction schemes. Top line, the FDR workflow needs the five steps to attain a super-resolution image. Bottom line, SDR is intrinsically simpler, which is only 3-step workflow. **(B)** The comparison of the mean execution time between different reconstruction schemes. The mean value of the execution time for SDR without deconvolution is up to 64 times faster than that of FDR.

In order to perform spectral processing of the images, Fourier transformation are needed with nine raw images (Figure 9A, top). After separating the spectra and OTF compensated, the spectra are recombined and enlarged isotropically. Then an inverse fast Fourier transform (iFFT) is used to form an SR image. The multiple operations of iFFT and FFT require significant CPU processing time. In contrast, only three simple steps operate in the SDR scheme, which are image multiplication, summation, and deconvolution (Figure 9A, bottom). The multiplied coefficient matrixes can be pre-calculated because they only depend on the sinusoidal light pattern. Consequently, SDR processes one SR image 7- to 64-fold more rapidly than FDR (Figure 9B).

**FIGURE 10 |:**
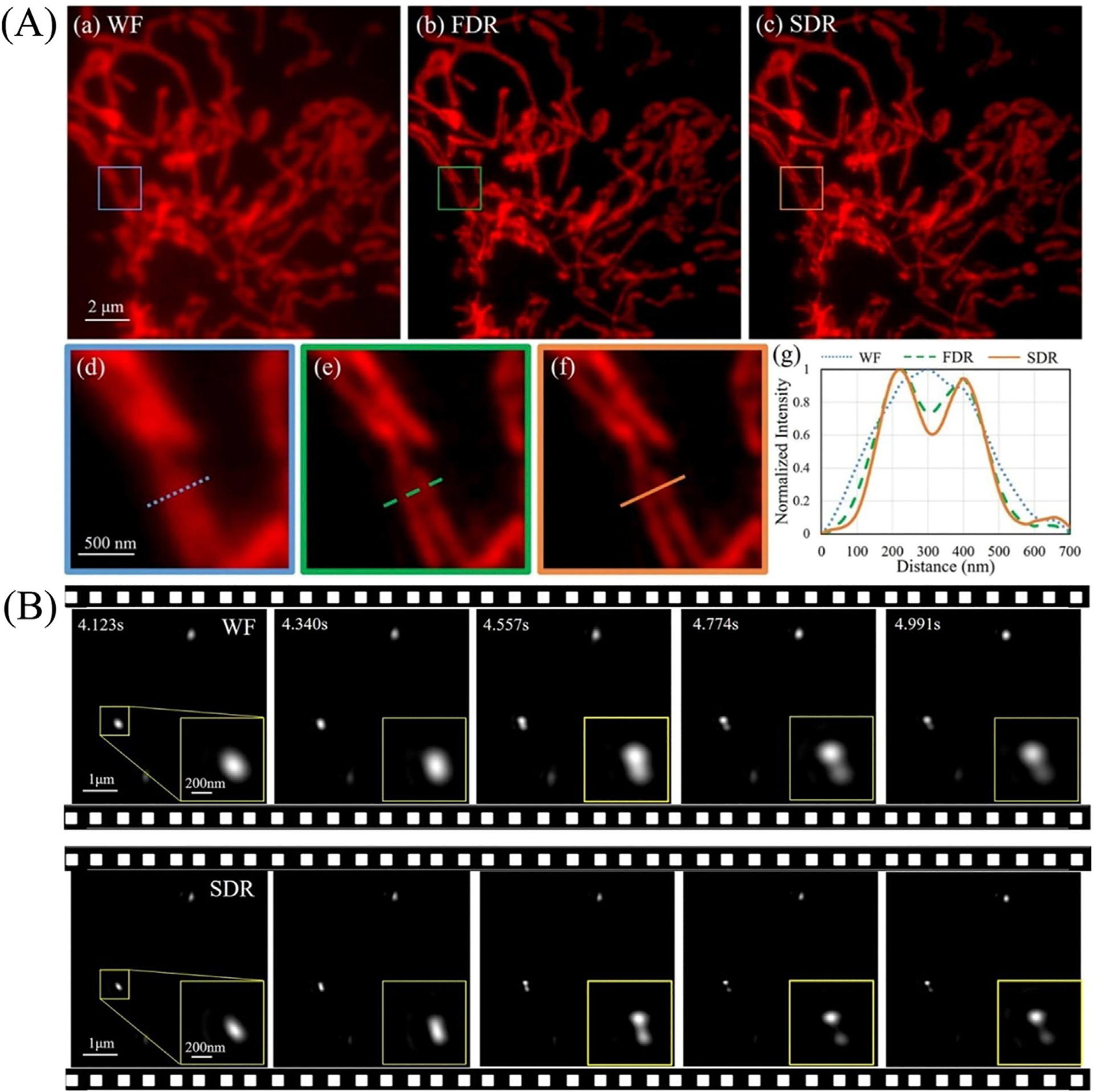
SDR resolves fine details better than FDR in both static and dynamic imaging [71]. (**Ai–iii**) Images reconstructed by wide-field, FDR- and SDR-SIM, respectively. (**Aiv–vi**) The magnified views in the boxed regions in (**Ai–iii**). (**Avii**) Intensity profiles along the marked lines in (**Aiv–vi**). **(B)** SDR enables the resolution of beads. Five time-sequential frames in an interval of 217 ms are shown. The zoom-in boxes display the relative position variance of two adjacent beads.

The superiority of SDR in producing SR images was demonstrated by imaging fluorescent cells and separately, 100 nm beads in solution (Figure 10). The results from cellular imaging show while both FDR and SDR reconstruction approaches produce comparable SR images, SDR is both faster and can discern fine details better than FDR. A further advantage of SDR is revealed when mobile fluorescent beads are imaged. In this example, the Brownian motion of the beads was recorded in real-time with nine raw images reconstructing one frame of the SR image. Importantly, only SDR was sufficiently rapid to produce these movies.

### Summary

Considering the growing array of SR-SIM reconstruction methods, picking the “right tool for the job” can be a challenging task. Nevertheless, some general trends become clear when a comparison of the techniques presented in this review is made (Table 2). This comparison is qualitative as the actual speed of each system is limited by many factors including the sample used for imaging, the microscope, and the computer hardware and software. As a reference, we use commercial SIM systems and compare acquisition and post-processing speed, and implementation complexity. For acquisition speed, 3-frame SIM and Deep learning methods perform the best and receive the most stars. This makes sense because less raw data are needed for SR image reconstruction and this results in better ways to track sample movement. Deep learning and SDR methods have a faster post-processing speed, which has the benefit of displaying reconstructed SR images of live-cells in real-time. However, these methods are more complex than the commercial ones, especially the deep learning method which requires thousands of pieces of raw data to build the deep neural network.

## CONCLUSION

As one of the super-resolution imaging techniques available, structured illumination microscopy has broken through the diffraction limit and become a powerful tool for studying living cells. As a result of low phototoxicity and high frame rate acquisition, SIM stands alone as the approach to imaging biological transactions at super-resolution and in real-time in living cells. Since its first appearance as an imaging modality 20 years ago, numerous approaches have been presented to increase acquisition speed and improve image reconstruction time. Further advancements in the optical layout such as those seen in fast SIM will further contribute to improvements in image acquisition and quality. The combination of GPU processing and the SDR algorithm will likely push image reconstruction speeds to even greater speeds. When these advances are combined with the use of brighter and more photostable dyes, the anticipated shorter exposure times during image acquisition will result in even lower phototoxicity and increases in imaging speed. Consequently, this will further establish high-speed SIM as the SR imaging choice for live-cell biological transactions in real-time.

## Figures and Tables

**FIGURE 6 |:**
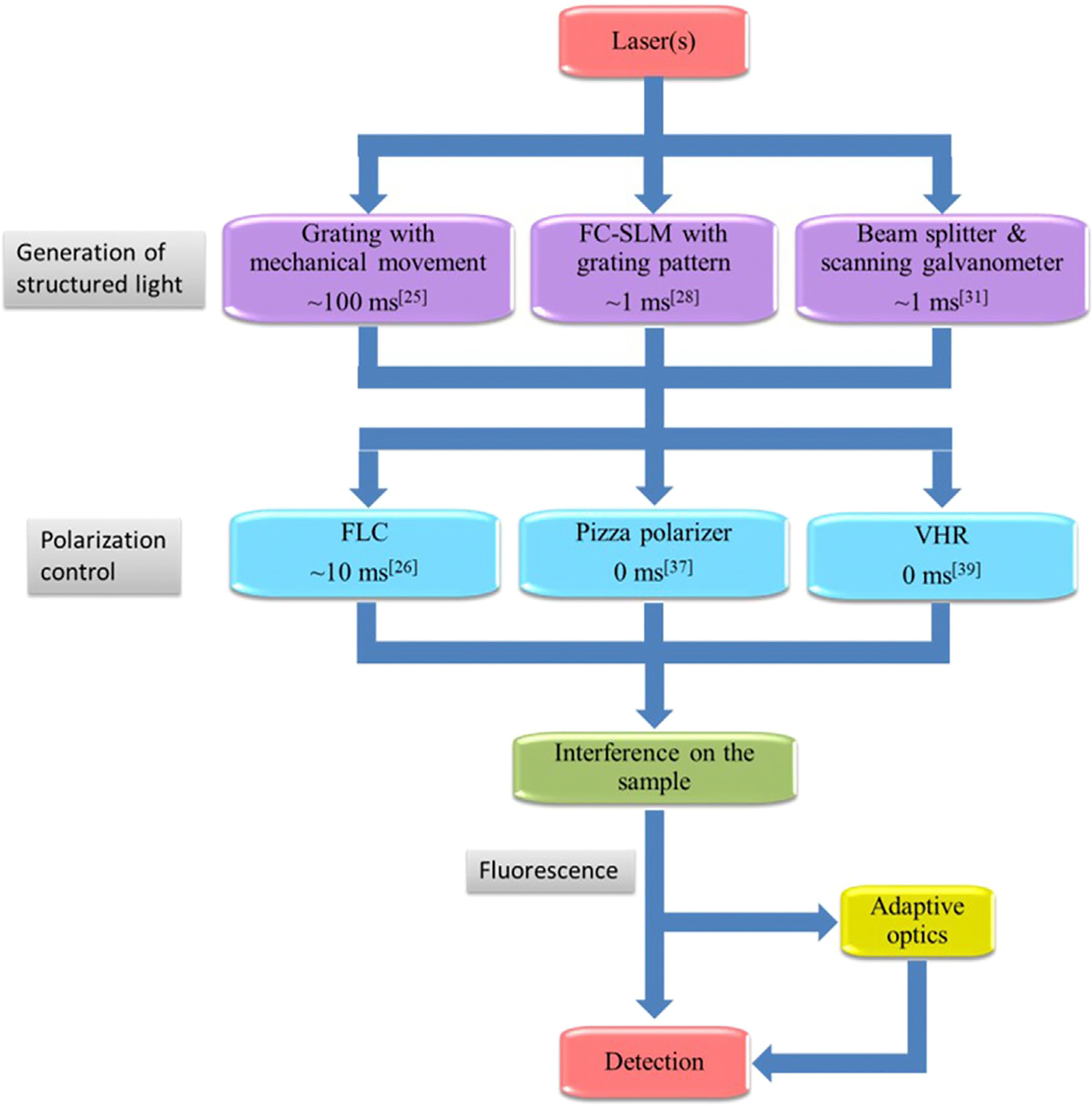
The flowchart for SIM. The majority of SIM systems illuminate samples with one or more lasers. The laser beam is then passed through a pattern-generating system, polarization control system, and interference on the sample. The fluorescence can be optimized by adaptive optics before being detected by the camera.

**TABLE 1 |:** Comparison of SIM performance.

Key component	FC-SLM [28]	Scanning galvanometer [31]	FLC [27]	Pizza polarizer [37]	Vortex half-wave retarder [39]	Rolling shutter cameras [44]
Objective	60 × NA1.2	60 × NA1.49	100 × NA1.49	63 × NA1.4	100 × NA1.49	63 × NA1.4
Frame rate	11 Hz@512 × 512 pixel	10 Hz@256 × 256 pixel	11 Hz@128 × 128 pixel	7.6 Hz@1024 × 768 pixel	162 Hz@512 × 100 pixel	79 Hz@512 × 512 pixel
Field of view	25 × 25 μm^2^	40 × 40 μm^2^	8 × 8 μm^2^	19.5 × 13.8 μm^2^	36 × 7.2 μm^2^	16.5 × 16.5 μm^2^
Excitation wave length	Multi	Multi	Single	Single	Multi	Single

**TABLE 2 |:** Comparison of reconstruction methods for fast SIM.

Component	Commercial	4-frame SIM	3-frame SIM	GPU	Deep Learning	SDR
Acquisition speed	★★★^a^	★★★★★	★★★★★★	★★★★	★★★★★★	★★★★
Post-processing speed	★★★	★★	★★	★★★★	★★★★★★	★★★★★
Implementing complexity	★★★	★★	★★	★★	★	★★★

a More stars indicate that the method performs better.
